# 79 Developmental Patterns of Objectively Measured Motor Competence and Musculoskeletal Fitness among Finnish Adolescents

**DOI:** 10.1093/eurpub/ckae114.058

**Published:** 2024-09-26

**Authors:** Timo Jaakkola

**Affiliations:** University of Jyväskylä, Faculty of Sport and Health Sciences, Jyväskylä, Finland

## Abstract

**Purpose:**

This study aims to examine the developmental patterns (rate of change) between motor competence (locomotor, object control, stability skills) and musculoskeletal fitness (upper body and abdominal) in a large sample of Finnish adolescents over four years. The previous evidence does not explicitly explore developmental patterns between musculoskeletal fitness and motor competence. Additionally, most previous studies have not examined specific aspects of musculoskeletal fitness (i.e., muscle groups), rather a focus on overall muscular fitness has been reported.

**Methods:**

Data was collected annually at five time points during scheduled physical education lessons. At baseline, 1147 (582 males, 565 females) Finnish adolescents aged 11.27 (0.33) years participated in data collection. Throwing-catching combination (object control), 5-leaps (locomotion) and side-to-side jumping (stability) tests were used to measure students’ motor competence. Curl-up (abdominal muscles) and push-up (upper body muscles) tests were used to analyze their musculoskeletal fitness. To answer the research question, a parallel latent growth curve model was implemented. The latent variables (Level, Slope) based on the observed variables with residuals (ε) were estimated.

**Results:**

Developmental changes (improvements) in push-ups were positively associated with improvements in all motor competency variables, demonstrating greater increases in upper body musculoskeletal fitness was related to greater increases for versatile motor skills (locomotor, object control, stability). Similarly, developmental changes in curl-ups were positively associated with improvements in 5-leaps and side-to-side jumping scores, demonstrating greater increases in abdominal musculoskeletal fitness was associated with greater improvements for locomotor and stability skills, but not for object control.

**Conclusions:**

It is likely that the relationship between individual motor skills and separate fitness variables is more complex given the multi-joint nature of motor skills and the inherent neuromuscular demand to perform with high effort in various physical activities and sports. For instance, at a neuromuscular level high levels of intra- and inter-muscular control and co-ordination are needed to effectively perform a range of fundamental movement skills. Thus, the development of musculoskeletal fitness and motor competence appear to be fundamentally linked, directly via neuromuscular function, and indirectly via psychosocial and behavioral mechanisms.

**Funding source:**

The Finnish ministry of education and culture funded this study.

